# Improved Masticatory Performance in the Partially Edentulous Rehabilitated with Conventional Dental Prostheses

**DOI:** 10.3390/medicina60111790

**Published:** 2024-11-01

**Authors:** Maria Angeles Lopez-Cordon, Laura Khoury-Ribas, Bernat Rovira-Lastra, Raul Ayuso-Montero, Jordi Martinez-Gomis

**Affiliations:** 1 Faculty of Medicine and Health Sciences, Department of Prosthodontics, School of Dentistry, University of Barcelona, 08907 Barcelona, Spain; marian.lopez@ub.edu (M.A.L.-C.); laura.khoury.ribas@ub.edu (L.K.-R.); brovira@ub.edu (B.R.-L.); jmartinezgomis@ub.edu (J.M.-G.); 2 Oral Health and Masticatory System Group, Bellvitge Biomedical Research Institute IDIBELL, L’Hospitalet de Llobregat, 08907 Barcelona, Spain

**Keywords:** fixed partial denture, masticatory system, mouth rehabilitation, partially edentulous, patient satisfaction, removable partial denture

## Abstract

*Background and Objectives*: Oral rehabilitation seeks to enhance mastication, a vital component of oral function that is compromised by tooth loss. This study aimed to assess the degree of improvement of masticatory performance in partially edentulous patients rehabilitated with removable partial dentures (RPD) or fixed partial dental prosthesis (FPDP). Changes in the occlusal contact area (OCA) and satisfaction with their chewing ability during the adaptation period were also evaluated. *Materials and Methods*: in total, 34 partially edentulous participants (median age 65.3 years; 56% women) who received an RPD or FPDP were assessed using masticatory performance assay, OCA calculation, and a visual analog scale (VAS). *Results*: Masticatory performance improved by 20% (range from 17% to 25%, *p* < 0.05) depending on the edentulism and the rehabilitation types. The OCA improved by 4.7 mm^2^ (*p* < 0.05) and satisfaction with the masticatory function improved by 9% (*p* < 0.05) 3 months after prosthesis insertion. *Conclusions*: Conventional prostheses benefited partially edentulous individuals, improving masticatory performance by 20%. Treatment also increased the OCA in all types of partial edentulism, except in Kennedy class I patients rehabilitated with RPD. Patients’ satisfaction with their chewing ability only increased in Kennedy class III patients rehabilitated with RPD.

## 1. Introduction

Tooth loss is a relatively common phenomenon that leads to poor oral function in adults who do not undergo prosthodontic treatment. It can affect speech, mastication, deglutition, social activities, and esthetics [1,2], including oral health-related quality of life (OHRQoL) [3,4]. Edentulous patients will often limit their food selection, preferring a soft diet [1,5,6]. Several factors, including age, polypharmacy, the use of removable dentures, missing teeth, low occlusal force, and low tongue pressure, and influence masticatory performance [7] with the loss of occlusal support being a key factor contributing to reduced performance [8]. The lack of occlusal support can be classified using the Kennedy classification, which distinguishes between bilateral free-end edentulism (Class I), unilateral free-end edentulism (Class II), posterior tooth loss bounded by adjacent teeth (Class III), and anterior tooth loss crossing the midline (Class IV) [9].

Oral rehabilitation aims to improve mastication [10]. Prosthetic treatment options for the partially edentulous include dental/implant-supported fixed prostheses and removable dental/implant prostheses [11]. Rehabilitation with dental implants can improve chewing efficiency and perceived masticatory capacity [12,13,14,15], using either fixed prostheses [11,15] or removable partial prostheses [16,17]. However, conventional dental prostheses (non-implant-supported) are still widely used and many patients with lower socioeconomical statuses remain edentulous due to the cost of implant-supported prostheses [18,19].

Some cross-sectional studies have associated tooth loss and the use of metal-clasp removable partial dentures (RPD) with reduced masticatory performance [7], while others have shown improved masticatory function compared with RPDs when using fixed partial dental prostheses (FPDP) anchored to adjacent teeth [20]. An objective measure of masticatory function is the masticatory performance calculated using the median particle size (MPS) [21] when comminuting an artificial test food (silicone tablets) [15,22,23,24]. This method has been used in subjects with natural dentitions [22,23] and in the partially edentulous [15,22,23,24]. Complementing this approach, visual analog scales (VAS) have been used to evaluate the subjective perception of masticatory function [15,25]. The occlusal contact area (OCA) is reported to be among the factors that most affect masticatory performance in totally dentate adults [22,26,27], while the number of restored teeth has been related to masticatory performance in the partially edentulous rehabilitated with different prosthetic options [15,28].

The main objective of this study was to determine the degree of masticatory performance improvement in partially edentulous patients rehabilitated with conventional prostheses (RPD or FPDP) in a single arch. As secondary objectives, (1) the variation in OCA after treatment and adaptation, (2) the variation in the satisfaction with their chewing ability during the adaptation, and (3) the factors associated with the degree of improvement in masticatory performance were evaluated. The null hypothesis stated that conventional prostheses (RPD or FPDP) would have no effect on masticatory performance in partially edentulous patients.

## 2. Materials and Methods

Participants were partially edentulous adults attending the Barcelona University Dental Hospital (HOUB) who were candidates for oral rehabilitation and did not accept implant-fixed partial prostheses (IFPP). The inclusion criteria were the unilateral or bilateral absence of at least one tooth in the posterior region, involving only one arch, and rehabilitated with conventional prosthesis (RPD or FPDP). Participants missing only one posterior tooth were invited to select FPDP or IFPP. The exclusion criteria were the need for vertical dimension changes, non-treated periodontal disease, orofacial pain, temporomandibular disorders and any restorative treatment planned in the 3 months after prosthetic placement.

Informed consent was obtained from all participants, and the study was approved by the local Clinical and Medicine Research Ethical Committee (CEIm-HOUB, Code 2017-13). All procedures were conducted between May 2017 and September 2022 following the principles of the Declaration of Helsinki and the STROBE (Strengthening the Reporting of Observational Studies in Epidemiology) guidelines.

### 2.1. Clinical Procedures

A full general, regional, and intraoral examination was conducted at diagnostic and recruitment appointments, including panoramic radiographs and dental casts. Participants who accepted the FPDP underwent dental preparation to obtain a full coverage bridge (porcelain fused to metal) between the two abutment teeth, and received a temporary restoration. Conventional techniques were applied to obtain each FPDP, which were adhered with glass ionomer cement. Participants who accepted an RPD underwent dental preparation to receive direct retainers (and, when appropriate, rests, proximal plates, and indirect retainers) to obtain a metal clasp-retained RPD using conventional techniques. Finally, all FPDPs and RPDs were adjusted with articulating paper to avoid undesirable eccentric contacts and interferences at the maximal intercuspal position. All participants were examined once a week after insertion, and adjustments were made until the prosthesis could be used comfortably.

### 2.2. Data Collection

Participant data, such as age, gender, and number of absent teeth, were obtained by clinical interview and examination. The type of edentulism was determined according to the Kennedy classification [9], and the available prosthetic support was classified as dental-mucosal support in Kennedy classes I and II, or as dental support in Kennedy class III. A freestyle masticatory performance analysis and an occlusal record were obtained at baseline (before dental preparations) and at 1 week and 3 months after inserting the prosthesis. In participants with RPD placement, the 1-week and 3-month follow-up were measured from the last appointment when they could comfortably use the prosthesis without additional adjustments.

**Figure 1:**
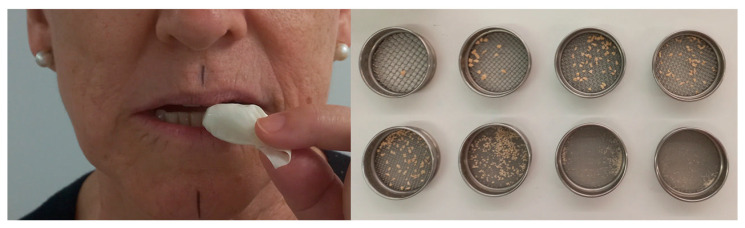
Participant initiating the masticatory assay and particles obtained on each sieve.

Masticatory performance was assessed in five tests in which participants chewed three quarters of a 2 g Optozeta tablet (Optosil P Plus, Heraeus Kulzer, Hanau, Germany; Zetalabor, Zhermack, Badia Polesine, Italy) measuring 5 mm thick and 20 mm in diameter, placed in a latex bag in a freestyle assay [15,23,24,29]. Particles obtained by the five tests (10 g) were dried and then sieved through eight sieves of 5.6 mm to 0.25 mm while being shaken for 1 min. The MPS was calculated using the weight distribution of the sieve contents and the Rosin-Rammler formula (Figure 1) [15,22,23,30].

**Figure 2:**
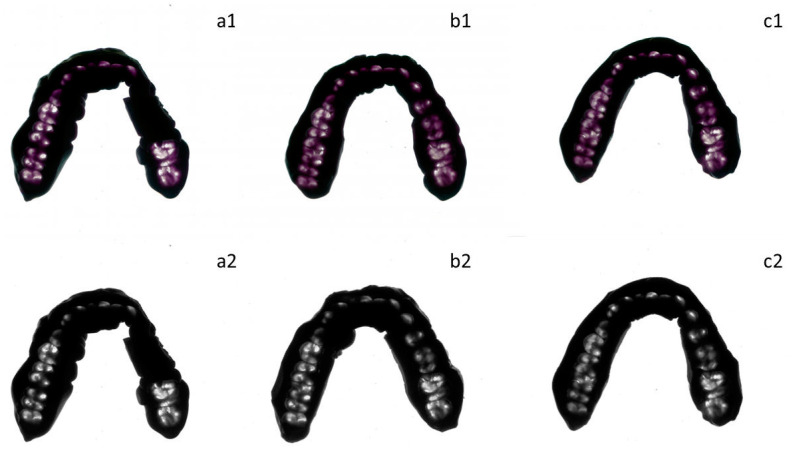
Occlusal record obtained from a participant with Kennedy class III treated with RPD. (**a1**) Record scanned at baseline and (**a2**) processed for OCA calculation; (**b1**) record scanned at 1 week follow-up and (**b2**) processed for OCA calculation; and (**c1**) record scanned at 3 months follow-up and (**c2**) scanned for OCA calculation.

The occlusal record was used to measure the OCA at the maximum intercuspal position [31,32]. The occlusal record material (Occlufast Rock, Zhermack, Badia Polesine, Italy) was injected on the occlusal surfaces of the mandibular teeth, covering the total arch surface, before participants closed their mouth in the intercuspal position by applying light force (participants were instructed to maintain this position at half of their maximum bite force) [33]. Once set, the material was removed, trimmed, scanned and analyzed using computer software (ImageJ v 1.52, National Institutes of Health), Figure 2. All interocclusal distances at 200 µm or less were considered in the calculation of the OCA, as this correlates with masticatory performance [22].

A 10-cm VAS was used to determine participant satisfaction with their chewing ability when using the prosthesis after 1 week and 3 months. They were asked the following question: “How satisfied are you with the prosthesis in terms of chewing ability?” on a scale from “completely dissatisfied (0%)” to “completely satisfied (100%).”

### 2.3. Sample Size Calculation

The sample size calculation was determined using the Granmo Sample Size Calculator, version 7.10 (Institut Municipal d’Investigació Mèdica, Barcelona, Catalonia, Spain), by accepting an alpha risk of 0.05 and a power of 0.8 in a two-tailed test to recognize a difference in one group of ≥0.66 mm in the MPS as statistically significant [15]. In total, 34 subjects were necessary, assuming a standard deviation of the difference of 1.3 and an estimated drop-out rate of 10% [22].

### 2.4. Statistical Analysis

The MPS, OCA, and satisfaction with their chewing ability values were not normally distributed (Kolmogorov–Smirnov test). Therefore, differences in the MPS and OCA from baseline to 1 week and 3 months after treatment were compared using Friedman’s two-way analysis of variance by ranks, adjusted by the Bonferroni correction; differences in satisfaction with the chewing ability from 1 week to 3 months after treatment were compared by the Wilcoxon signed-rank test. The percentage of changes in MPS, OCA, and satisfaction were calculated to test possible correlations with clinical factors (Kennedy class and prosthetic support) or treatment-related factors (prosthetic type and number of teeth restored). The Kruskal–Wallis test or Spearman correlation were used (when appropriate) to determine the relation between MPS at 3 months and the percentage improvement in MPS as dependent variables, with Kennedy class, prosthetic support, prosthesis type, total number of teeth restored, and OCA or satisfaction changes as independent variables.

Finally, a multiple linear regression model was performed using a stepwise forward method to examine whether the examination point (baseline, 1 week after treatment, and 3 months after treatment), prosthetic support, and type of prosthesis significantly explained the changes in freestyle masticatory performance.

## 3. Results

Overall, 38 participants enrolled in the study and four did not attend all appointments; therefore, 34 adults (19 women and 15 men) with a median age of 65.3 years old completed the study. Of these, six were classified as Kennedy class I, 11 as class II, and 17 as class III (7 with RPD and 10 with FPDP). Thus, 17 participants had dental-mucosal support for the prostheses and 17 had dental support. The baseline clinical characteristics of participants are described in Table 1.

### 3.1. Masticatory Performance

**Figure 3:**
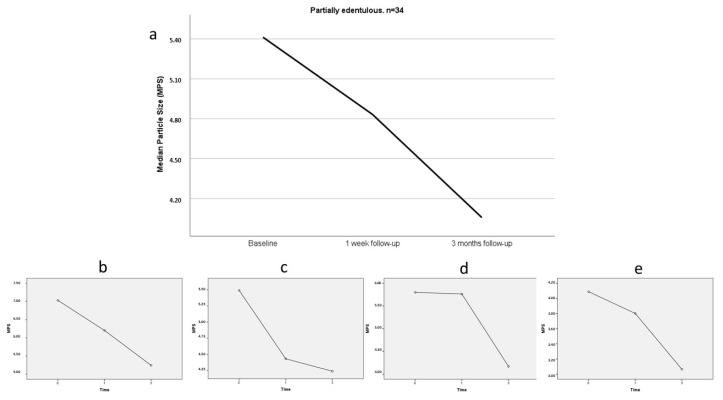
Variation in masticatory performance at different evaluation time points, expressed in Median Particle Size (MPS), where a lower MPS indicates better masticatory performance. (**a**) Partially edentulous; (**b**) Kennedy class I; (**c**) Kennedy class II; (**d**) Kennedy class III; (**e**) Kennedy class IV. 0 = Baseline; 1 = One-week follow-up; 3 = Three-month follow-up.

The baseline MPS in the freestyle mastication assay for partially edentulous participants was 5.41 mm, which reduced significantly to 4.06 mm at 3 months (20.1% reduction in MPS, *p* < 0.05 by Friedman’s test), regardless of the type of edentulism and Kennedy classification. Kennedy class I participants improved masticatory performance significantly during the 3-month adaptation period (25.1% reduction in MPS, *p* < 0.05 by Friedman’s test). The MPS values and differences at the different assessments are detailed in Table 2, and graphically represented on Figure 3.

### 3.2. Occlusal Contact Area

At 3 months, the OCA improved by a mean of 4.67 mm^2^ (±7.15) in the partially edentulous (*p* < 0.05 by Friedman’s test). By Kennedy class, the OCA reduced by 1.52 mm^2^ (±6.96) in class I and increased by 4.26 mm^2^ (±6.75) and 4.35 mm^2^ (±5.28) in classes II and III rehabilitated using an RPD; it also increased by 7.25 mm^2^ (±8.77) in class III rehabilitated using FPDP (Table 3).

### 3.3. Patient Satisfaction

All subgroups except participants with Kennedy class I experienced improved satisfaction with chewing ability using the prostheses during the adaptation period; however, these differences were statistically significant only for Kennedy class III rehabilitated using RPD (*p* < 0.05, Wilcoxon signed-rank test) (Table 4).

### 3.4. Factors Related to the Improvement in Masticatory Performance

MPS at 3 months was related to Kennedy class (*p* < 0.003), prosthetic support (*p* < 0.024), and prosthetic type (RPD or FPDP) (*p* < 0.001, Kruskal–Wallis tests by ranks). The percentage improvement in MPS was positively correlated to the improvement in satisfaction with their chewing ability (*p* < 0.001, Spearman correlation test). Stepwise linear regression analysis indicated that the examination point (i.e., baseline, 1 week after treatment, and 3 months after treatment) (*p* < 0.001) and the combination of examination point and type of prosthetic support (i.e., dental-mucosal or dental) (*p* < 0.05) were associated with the changes in MPS during the freestyle assay.

Finally, Table 5 summarizes the principal findings of the masticatory assay, occlusal contact area, and satisfaction evaluations.

## 4. Discussion

These results suggest that treating partial edentulism using a conventional prosthesis (i.e., RPD or FPDP) improves masticatory performance in a freestyle assay. Therefore, the null hypothesis was rejected. The mean improvement was 20%, ranging from 17% in Kennedy class II to 25% in Kennedy class I. The observed improvement was correlated with the improvement on the chewing ability perceived by the patients.

The partially edentulous (including all edentulism and rehabilitation types) achieved a mean reduction in the MPS value from 5.41 mm at baseline to 4.06 mm after rehabilitation. This final value is comparable to results for a partially edentulous group missing two natural teeth under the same conditions [15]; the Kennedy class III subgroup rehabilitated with FPDP also obtained a final MPS value similar to participants with natural teeth [15]. Furthermore, the MPS at 3 months in this study was found to be associated with the Kennedy class, prosthetic support, and prosthetic type. These results highlight the importance of maintaining the posterior teeth and of educating the general public about the need for proper dental care to preserve Kennedy class III partial edentulism, so as to allow dental support and FPDP as a treatment option. The Kennedy class II group who were not candidates for FPDP and did not show improved masticatory performance with an RPD should be advised to opt for implant-supported rehabilitation.

The partially edentulous patients experienced a significant improvement in masticatory performance, but needed the 3-month adaptation period to obtain this improvement. This adaptation period is longer than the 2-month period used in other studies of masticatory function [34,35] but has previously been used to test masticatory performance [15]. During the freestyle mastication assay, participants could move the bolus from one side to the other, and so the Kennedy class II group (unilateral dental prosthetic support) and Kennedy class III group (bilateral dental prosthetic support) could use the most effective side. This may explain the non-significant effect of prosthetic use on masticatory performance. By contrast, the Kennedy class I group have a bilateral dental-mucosal prosthetics support, and experienced changes in masticatory performance on both sides. Nevertheless, the freestyle mastication assay more closely reproduces the true chewing situation [23].

The OCA improved by a mean of 4.67 mm^2^ (±7.15); however, this improvement included all three Kennedy classes, including the reduction in class I. Moreover, the OCA did not improve immediately after treatment in any patient over the adaptation period needed to show improvement. Although the method used to evaluate the OCA is considered the most valid and reliable [32], Lu et al. have shown that the OCA increases when heavy forces are applied, regardless of whether there are missing teeth [36]. Consequently, although participants were instructed to maintain a light force for the occlusal record, differences in prosthetic support probably affected the ability to maintain this force, especially after placing a bilateral dental-mucosal RPD (i.e., Kennedy class I). This improvement was not significant after treatment in Kennedy classes II and III, indicating that the partially edentulous group rehabilitated with a conventional prosthesis probably need the adaptation time to record improvements in the OCA. Neither the MPS value at 3 months nor the improvement in MPS was associated with the change in OCA, consistent with the results of a previous study in participants with similar characteristics rehabilitated with IFPP [15]. Although cross-sectional studies suggest that the OCA is an important factor in masticatory performance in dentate adults [22,26,27], this study found that Kennedy class, prosthetic support, and prosthetic type were the main factors related to masticatory performance in the partially edentulous rehabilitated with conventional prostheses.

Satisfaction with chewing ability only improved in the Kennedy class III group rehabilitated with RPD, with changes in satisfaction related to improved masticatory performance during the adaptation period. Interestingly, satisfaction fell slightly in the Kennedy class I subgroup with improved masticatory performance. Satisfaction is a subjective value that may be influenced by other factors, such as patient expectations and prosthesis limitations; this subjectivity may explain the consistent evidence showing no correlation between patient satisfaction and masticatory performance [15,16,37].

This study has several limitations. Although the sample size was sufficient to detect masticatory improvement in the partially edentulous, the findings of the regression analysis should be interpreted with caution due to the small number of participants in subgroups with different prosthetic supports or types. The lack of randomization, inherent to the study design, along with the inclusion of different types of edentulism, may obscure potential confounding factors. Additionally, the muscular force was not controlled at the different time points of masticatory performance assessment, with participants only instructed to maintain half of their maximum bite force when setting the occlusal records. However, to homogenize the procedures under similar conditions, the assessment was performed at 1 week and 3 months after the appointment when the prostheses were reported to fit comfortably. Finally, despite the wide use of comminution of an artificial food to assess masticatory function, the results obtained in this study must be applied with caution to other food types. Future investigations adding comparisons between subgroups with a larger sample size, and the use of a two-colour chewing gum to assess the masticatory mixing ability would complement the results of the present study [20,38].

## 5. Conclusions

Conventional prostheses improve masticatory performance by 12 to 28% in the partially edentulous. Treatment with conventional prostheses increased the occlusal surface and the patients’ satisfaction with their chewing ability after three months. Although all types of conventional prostheses provide a similar percentage of masticatory performance improvement, the final masticatory performance may depend on the type of initial edentulism.

## Figures and Tables

**Table 1:** Participant characteristics.

Group	n	Age	No. Absent Teeth	No. Teeth Restored
Partially edentulous	34	65.3 (11.1)	5.2 (4)	4.2 (2.3)
Kennedy class I	6	65.6 (8.6)	8.5 (6.9)	6.2 (1.7)
Kennedy class II	11	65.3 (11.7)	5.4 (1.9)	5.4 (1.4)
Kennedy class III	17	65.2 (12)	3.9 (3.3)	2.6 (1.9)

Data show the mean (standard deviation) of the clinical and prosthetic factors. Abbreviation: No., number.

**Table 2:** Masticatory performance in a freestyle assay on the different study times.

Group	MPS (mm)				% MPS Reduction at 3 Months (95% CI)
	n	Baseline	1 Week FU	3 Month FU	
Partially edentulous	34	5.41 (2.3)	4.83 (2.1)	4.06 (1.6) ^ab (*p* < 0.001)^	20.1% (12.22 to 28.03)
Kennedy class I (RPD)	6	7.03 (1.1)	6.20 (1.82)	5.24 (1.1) ^a (*p* = 0.028)^	25.1% (11.41 to 38.70)
Kennedy class II (RPD)	11	5.49 (2.52)	4.43 (1.83)	4.24 (1.98)	16.8% (−3.04 to 36.61)
Kennedy class III (RPD)	7	5.8 (2.41)	5.76 (2.45)	4.17 (1.32)	22.6% (−0.02 to 45.17)
Kennedy class III (FPDP)	10	4.09 (0.63)	3.8 (0.5)	3.06 (0.26)	19.1% (5.05 to 33.23)

In participants with RPD, the 1-week and 3-month follow-up were measured from the last appointment when they could comfortably use the prosthesis without additional adjustments. Significant differences (*p* < 0.05) by the Friedman test adjusted by the Bonferroni correction for multiple tests: a from baseline as a reference; b from after treatment to 3 months. Abbreviations: CI, confidence interval; FPDP, fixed partial dental prostheses; FU, follow-up; RPD, removable partial dentures. MPS is reported as mean (standard deviation).

**Table 3:** Occlusal contact area in mm^2^.

Group	n	Baseline	1-Week FU	3-Month FU
Partially edentulous	34	12.14 (7.48)	12.69 (6.7)	16.82 (7.8) ^ab (*p* < 0.001)^
Kennedy class I (RPD)	6	12.71 (5.72)	6.78 (3.39)	11.77 (4.68)
Kennedy class II (RPD)	11	11.19 (7.36)	12.05 (6.74)	15.45 (8.3) ^a (*p* = 0.038)^
Kennedy class III (RPD)	7	11.23 (5.83)	12.26 (5.26)	15.58 (4.56) ^a (*p* = 0.018)^
Kennedy class III (FPDP)	10	13.70 (10.14)	16.48 (7.38)	20.96 (8.94) ^a (*p* = 0.002)^

Data show the mean (standard deviation) of the occlusal contact area. In participants with RPD, the 1-week and 3-month follow-up were measured from the last appointment when they could comfortably use the prosthesis without additional adjustments. Significant differences (*p* < 0.05) by Friedman’s test adjusted by the Bonferroni correction for multiple tests: a from Baseline as a reference; b from After treatment to 3-month follow-up. Abbreviations: FPDP, fixed partial dental prostheses; FU, follow-up; RPD, removable partial dentures.

**Table 4:** Degree of satisfaction with the chewing ability. Visual analog scale punctuation. Mean (SD).

Group	n	1 Week FU	3 Month FU
Partially edentulous	34	8 (2.28)	8.91 (1.81) * ^(*p* = 0.006)^
Kennedy class I (RPD)	6	8.04 (2.98)	7.88 (1.86)
Kennedy class II (RPD)	11	7.71 (2.02)	8.68 (2)
Kennedy class III (RPD)	7	6.44 (2.16)	8.34 (1.61) * ^(*p* = 0.018)^
Kennedy class III (FPDP)	10	9.26 (1.79)	9.99 (1.42)

Data show the mean (standard deviation) of the visual analog scale. In participants with RPD, the 1-week and 3-month follow-up were measured from the last appointment when they could comfortably use the prosthesis without additional adjustments. * Significance on Wilcoxon test: *p* < 0.05. Abbreviations: FPDP, fixed partial dental prostheses; FU, follow-up; RPD, removable partial dentures.

**Table 5:** Reduction in MPS (mm), changes in occlusal contact area (mm^2^) and in the degree of satisfaction with the chewing ability between baseline and the 3-month follow-up. Mean (SD).

Group	n	MPS Reduction	OCA Variation	Satisfaction Variation
Partially edentulous	34	1.36 (1.5)	4.68 (7.2)	0.92 (1.9)
Kennedy class I (RPD)	6	1.79 (1)	−0.94 (7)	−0.16 (2.3)
Kennedy class II (RPD)	11	1.25 (1.7)	4.26 (6.8)	0.97 (1.7)
Kennedy class III (RPD)	7	1.63 (1.7)	4.35 (5.3)	1.9 (1.8)
Kennedy class III (FPDP)	10	1.02 (1.4)	7.25 (8.8)	0.73 (2)

## Data Availability

The data presented in this study are available on reasonable request to the corresponding author.
